# Supplementary figures and images for: Multimodal Diabetes Empowerment for Older Adults with Diabetes
Source: Int J Environ Res Public Health. 2022 Sep 8;19(18):11299. doi: 10.3390/ijerph191811299 (PMC9517437; doi:10.3390/ijerph191811299)

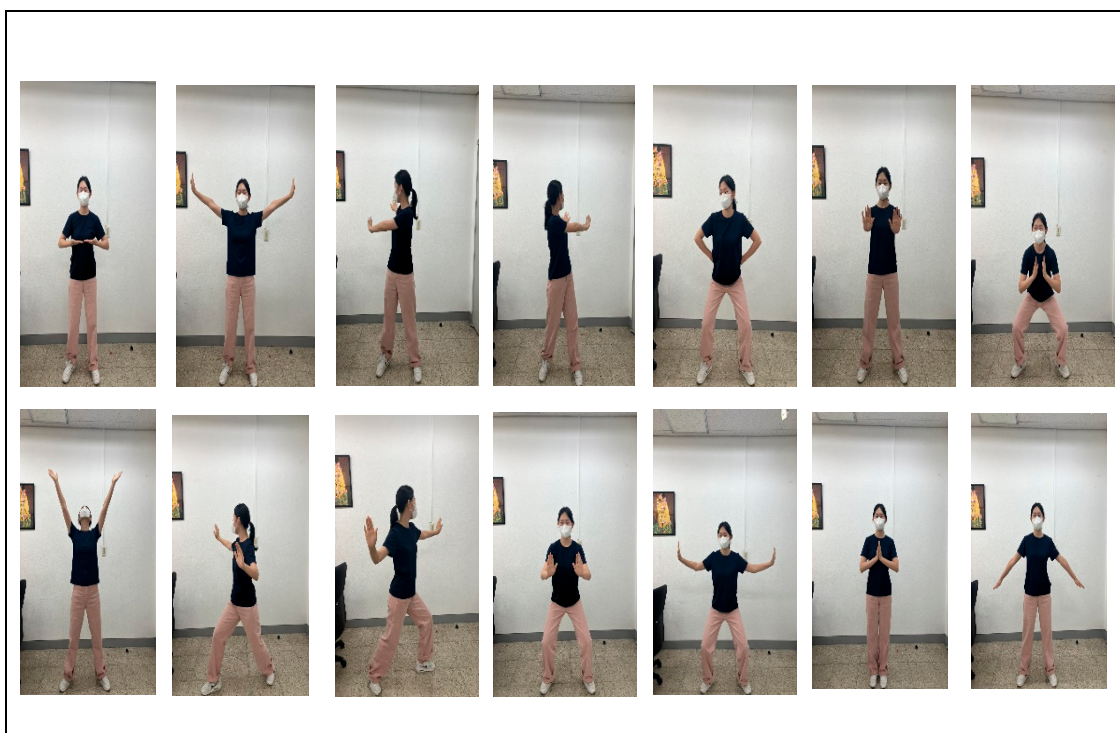

Figure S1: The pictures of BeHaS exercise of this Dia-Empower program

Supplement: Supplementary file 1 [file ijerph-19-11299-s001.zip › ijerph-1877228-supplementary.pdf]
